# Perceived training needs by tutors of family and community medicine and nursing of specialized health training: a qualitative descriptive study

**DOI:** 10.3389/fmed.2024.1329671

**Published:** 2024-12-16

**Authors:** Sofía Berlanga-Fernández, Miriam Rodríguez-Monforte, Rosa M. Pérez-Cañaveras, Alicia Valer-Martínez, Silvia Copetti-Fanlo, Pere J. Simonet-Aineto, Flores Vizcaya-Moreno, Rosa Villafáfila-Ferrero

**Affiliations:** ^1^Unidad Docente de Atención Familiar y Comunitaria Costa Ponent, Institut Català de la Salut, Barcelona, Spain; ^2^Grup de Recerca en Docència a l'Atenció Primària (GREDOCAP), Fundació Institut Universitari per a la recerca a l'Atenció Primària de Salut Jordi Gol i Gurina (IDIAPJGol), L'Hospitalet de Llobregat, Barcelona, Spain; ^3^Global Research on Wellbeing (GRoW), Facultat de Ciències de la Salut Blanquerna, Universitat Ramon Llull, Barcelona, Spain; ^4^Grupo de Investigación Enfermería Clínica (EC), Facultad de Ciencias de la Salud, Universidad de Alicante, Alicante, Spain; ^5^Gerència Territorial Metropolitana Sud, Institut Català de la Salut, Barcelona, Spain

**Keywords:** primary health care, clinical mentors, clinical tutors, specialized medical and nursing training, mentorship

## Abstract

**Introduction:**

Specialized Health Training is a postgraduate training pathway in which physicians and nurses can choose to continue their learning and obtain the qualification of specialist professional in a specific field. The training is eminently practical with different clinical tracks in which nurses and physicians are tutored by clinician tutors. Our research aims to describe the experiences and perceptions of clinician tutors related to their own teaching performance and training needs.

**Methods:**

We conducted a descriptive qualitative study. The sample consisted of active clinician tutors of specialized health training of family and community nursing and medicine, this being the main inclusion criterion, regardless of the number of years of experience as a tutor. Data were analyzed following a content analysis method.

**Results:**

Four focus groups were held with 25 participants: 32% nursing tutors (8) and 68% tutors of medicine (17). The main categories identified were: (1) teaching performance; (2) training needs; and (3) characteristics of an ideal training program. The sample consisted of a majority of tutors of medicine compared to nursing.

**Conclusion:**

Clinician tutors of Specialized Health Training of Family and Community nursing and medicine express the need to acquire pedagogical tools, to develop communication skills and to create a tutor network in order to improve their mentorship practice. Furthermore, more institutional recognition and protected time are also highlighted as important elements for their mentorship role. The findings of our research can serve as a guideline to start designing a training plan that meets the real needs of clinician tutors.

## 1 Introduction

Specialized Health Training is a postgraduate educational pathway in Spain in which health professionals can choose to continue their learning after graduation and obtain the qualification of specialist professional in a specific field. In the case of physicians and nurses, to access this pathway, a state examination must be passed (1). The specialty of family and community nursing or medicine is one of the postgraduate options to choose, offering the highest number of places in Spain for the training of future specialists. It is developed through a 4 year-postgraduate course in the case of physicians and a 2-year postgraduate course in the case of nurses (2).

The postgraduate training is led and carried out through Teaching Units spread throughout Spain and is eminently practical with different clinical tracks. During their training, nurses and physicians develop knowledge, attitudes, and skills toward the diagnosis, treatment and prevention of diseases at all stages of life, as well as in health promotion. The program includes rotations in different health services related to the content of the training program. Emphasis is placed on teamwork and patient, family and community centered care. Upon completion, nurses and physicians obtain a specialty diploma, allowing them to practice in Primary Healthcare. In addition, research and continuing education are encouraged (3, 4). During their training, nurses and physicians are tutored by clinician tutors. Clinician tutors are nursing and medicine professionals with demonstrable clinical experience and who require teaching accreditation to exercise their role. The accreditation can be achieved through the fulfillment of a series of requirements related to clinical-care, research and teacher profile. Being a clinician tutor entails having a deep knowledge on the training program and teaching methodologies for the training of future nursing and medicine specialists (4–7). Therefore, the great diversity of teaching methodologies requires clinician tutors to train to develop their role optimally, so that they can learn to use such methodologies in accordance with real practice and with the specific activities of the different environments in which the student practices (6–10). However, clinician tutors are often undertrained, raising concerns about the quality of mentoring support. Literature has highlighted there is a lack of robust mentoring training programs to ensure that mentors are trained in best practices around mentoring tools and techniques (11, 12). Recent evidence has shown improvement in mentors' and mentees' perceptions of mentor competence following structured, formalized training on best practices in mentoring (13). Some examples of courses aimed at training clinician tutors (14, 15) have shown an increase in the knowledge and skills of tutors and also better ratings from their mentees (16).

Paradoxically to these positive outcomes, there is evidence that suggests that the infrastructure and support for the mentoring role is not always adequate. The need for training comes into conflict with constant clinical commitments and a lack of time to get access to tutor-training courses. Also, there is a high degree of variability in the content of the teaching programs or the hours of dedication required for tutoring and a lack of support networks for mentors to develop within their role. National and international literature supports the need to explore and define the specific content of a training program aimed at enhancing the competence of clinician tutors that is sensitive to the challenges they face in their day to day practice, considering a participative approach (17–19). Some authors have highlighted there is a need to approach the real training needs of clinician tutors and generate accessible training (18), considering the tutoring methodologies to build a realistic, adapted tutor-training plan (20). Active participation in the design of teaching plans by the recipients of such plans is an important aspect to consider for their transferability and impact on the professional and personal development of future specialists (21).

To date, no study has described the experiences in relation to the core elements for good teaching performance perceived by the clinician tutors of nursing and medicine specialized health training in the field of family and community nursing and medicine. For all the above reasons, and considering the importance of the teaching competence of clinician tutors of specialized health training, and the lack of a realistic approach to their vision on the performance of their teaching role, this study aims to describe the experiences and perceptions of clinician tutors related to their own teaching performance and training needs.

## 2 Methods

### 2.1 Design

The current descriptive qualitative study enabled exploring clinician tutors' experiences with their teaching role, and perceptions, needs and barriers to their own training. Consolidated Criteria for Reporting Qualitative Research (COREQ) were used to enhance its quality and transparency (22).

### 2.2 Sample

A purposive sample was used consisting of active clinician tutors of medicine and nursing of postgraduate training on Family and Community Care in Catalonia, Spain, this being the main inclusion criterion, regardless of the number of years of experience as a tutor. The type of sampling was intentional and homogeneous with respect to the representation of different primary care centers where clinician tutors develop their clinical practice, who gave consent to participate in this study. Potential participants were contacted by email by the first author. The participation in the study was also proposed in a work meeting in which there was a representation of tutors from different healthcare centers. In this meeting the objective of the study was explained as well as the research methodology and the tools and information that was planned to be gathered. Those tutors who voluntarily decided to participate in the research wrote an email to the research team. The sample size was conditioned by data saturation.

### 2.3 Setting

The postgraduate training on Family and Community Care of nurses and physicians is developed in Teaching Units spread throughout Spain and is eminently practical with different clinical tracks. Our study includes one Teaching Unit which involves 24 Primary Healthcare centers in which there are 216 tutors. Each nursing or physician postgraduate student is allocated in one Primary Healthcare center and assigned one tutor.

### 2.4 Participants

A total of 25 tutors participated in the study: eight nursing tutors and seventeen tutors of medicine (Table 1).

**Table 1:** Description of the study population and context of data collection.

**FG number**	**Number of participants**	**Women**	**Men**	**Nursing Tutor**	**Medicine Tutor**	**Duration (hours)**	**Location**
1	4	4 100%	0	2 50%	2 50%	1:01	Online
2	7	4 57.14%	3 42.86%	2 28.58%	5 71.42%	1:10	Online
3	8	2 25%	6 75%	1 12.5%	7 87.5%	1:28	Online
4	6	6 100%	0	3 50%	3 50%	1:16	Online

FG, Focus Group.

Of the clinician tutors of the Family and Community Nursing postgraduate training, six were in their 1^st^ year as tutors, while two had been tutoring students for more than 5 years. In the case of the Family and Community Medicine postgraduate training, three tutors were in their 1^st^ year as clinician tutors, while 14 had been tutoring students for more than 5 years.

### 2.5 Data collection

Four focus groups were developed from November of 2021 and March of 2022. Each session lasted between 60 and 90 min, and was led by a moderator and an observer, both authors of this article, who promoted group conversation by means of a script with a series of structured questions related to the bibliography consulted (Table 2). They recorded their impressions, annotations, and reflections individually and being faithful to the experiences and meaning that the participants gave to the study phenomenon. Given the epidemiological situation of the COVID-19 pandemic, the focus groups were held virtually and were video-recorded with the permission of the participants. The focus groups were held in Spanish.

**Table 2:** Focus group script.

• As a tutor, what teaching methodologies do you use in tutoring residents? • As a tutor, what are the reasons for using one or another teaching methodology? • As a tutor, what training needs do you have? • What is your opinion regarding whether there are barriers to accessing tutor training? • As a tutor, what would you include in a training plan aimed at tutors? • Would you like to add more information/ideas?

The leader (primary care physician) and observer (primary care nurse) of the focus groups were part of the research team. Both had been tutors and knew the clinician tutors through professional connections.

### 2.6 Data analysis

The text arising from the focus groups was the reference universe of the participants and, through successive readings, it enabled capturing the various elements that make up their significant world and their vision of the phenomenon. The regulatory criteria to ensure methodological rigor took into account the suitability of the methodology, relevance, validity, and reflexivity (22). The researchers that conducted the analysis are Family and Community Medicine and Nursing experts who work closely with clinician tutors in the development of the postgraduate training of future medicine and nursing specialists in the field of Family and Community Healthcare.

The sessions were transcribed verbatim and analyzed following the content analysis method (23). As Bardin defines, content analysis is a technique designed to obtain, by means of systematic and objective procedures of the content description of the messages, indicators (quantitative or not) which enable the inference of knowledge regarding the production and reception conditions (24).

The analysis focused on explicit content, so the degree of interpretation was low. The analysis phases were: (1) Pre-analysis: transcription of the material, general reading and delimitation of what was analyzed; (2) Exploration of the material: definition of codes (minimum units of meaning in relation to the objective of the study), identification of the relationship between code and interpretation of categories and subcategories; (3) Processing of the results and interpretation. The condensation of information for the analysis was inferential, using intuition, reflective and critical analysis.

The process developed for the analysis was conducted as follows. First, transcripts were read and re-read in order to become familiar with the data. Then data from the focus groups was analyzed by researchers SF and RV, generating the initial coding. This analysis was performed with the support of ATLAS.ti 8, and no discrepancies in coding were noted (25). Data was analyzed to develop preliminary categories. The researchers reviewed the categories and discussed their relevance in relation to the data. Categories were then defined and named. Any discrepancies were resolved through discussion. Finally, the report of the findings was produced which was shared with the focus group participants for their review and approval. The development of the focus groups and the analysis process was conducted in Spanish. Moreover, the translation of the quotations was developed by a professional translator reassuring the rigor of the whole process.

This study was approved by the Institutional Ethics Committee (code 4R 21/011).

## 3 Findings

Our findings describe the identification of three overarching categories emerging through content analysis that capture the experiences and perceptions of the teaching performance of clinician tutors, but also their needs for their own training in order to ensure an optimal development of their role. The categories include: (1) teaching performance; (2) training needs; and (3) characteristics of an ideal training program.

### 3.1 Teaching performance

The category teaching performance entails the description of the teaching strategies, the factors that determine the prioritization of the teaching strategies, and the challenges that the clinician tutors encounter within the development of the teaching-learning process. Three subcategories were identified: teaching strategies; prioritization of the teaching strategies; and barriers to teaching performance.

#### 3.1.1 Teaching strategies

An heterogeneous set of teaching strategies were used among the interviewed tutors. The most widely used were traditional strategies applied in the clinical learning environment such as feedback, observation, clinical case and problem resolution, reflection, critical incidents, and clinical sessions.

“*I usually use feedback; I try to give feedback based on a specific situation, case, conflict, something that has happened in the primary healthcare center…I ask the students: what would you change; what would you do if you confronted the same situation in the future…” (tutor 7)*.

Likewise, peer learning, video-recording or gamification were also highlighted. These stand as new approaches that have evolved in the past years due to the characteristics of Generation Z students which demand a more virtual learning environment and linking mentorship learning to clinical experiences.

“*We use videorecording as a system. The students like it and have generally continued to do so” (tutor 12)*.

“*We organized a teaching seminar based on projects and gamification teaching experiences” (tutor 23)*.

#### 3.1.2 Prioritization of the teaching strategies

Some tutors reflected on the importance of knowledge building for deciding which teaching strategies they would prioritize. Many postgraduate students, especially nurses, when they start their postgraduate degree, have already worked in clinical practice, so it is important to unlearn some aspects and give space to integrate new knowledge. The process of becoming a specialist not only entails the acquisition of knowledge and skills, but also of attitudes and the opportunity to grow as a professional.

“*One question is what we want to teach the students […] sometimes the resident has to be able to unlearn in order to learn […]” and “Students with a very high level of knowledge are being produced, but the residency is an opportunity for professional growth […] aspects of attitude, know-how, knowing how to be need to be worked on” (tutor 17)*.

The fact that learning is something that moves beyond the generation of new knowledge and skills facilitates the expression of the main reasons that determine the teaching strategies to apply by the clinician tutors: the learning needs/style of the student and the tutor's previous experience. Moreover, teaching strategies also need to consider the achievement of the learning outcomes described in the official training program. Thus, flexibility must always be present when deciding what/how to teach each day.

“*What I'm most worried about now is to be training people. Knowing how (to) get it right. Focus on the needs of the student. Each person is different […] How you work on the most emotional ” (tutor 13)*.

A second tutor also highlighted:

“*There are people who are more theoretical, different styles, for example, what works for me is to review patients, their records well reviewed […] but what works for me might not work for the student […]” (tutor 1)*.

Despite the responsibility that entails the performance of a clinician tutor, their training is not mandatory. Consequently, according to our informants, the experience gathered along the years substantially conforms, in many cases, the foundation of their pedagogical decisions.

“*I think it depends on the moment, there are tutors who have a greater affinity with one teaching method, because they have worked more on them and for whom it's more second nature” (tutor 9)*.

“*We apply what we know based on our experience” (tutor 20)*.

#### 3.1.3 Barriers to teaching performance

The main barriers expressed by our informants for the development of their role as tutors were the lack of time and the lack of organization of the healthcare center around the training of students. Primary healthcare centers' main aim is to provide clinical assistance to patients and families. Therefore, they are not designed around the learning needs of the students.

“*The agenda is not adapted to when the student is there, the pressure of healthcare makes it all difficult” (tutor 21)*.

### 3.2 Training needs

The tutors expressed the need to look for new ways of teaching and maintain their ability to learn and to develop as professionals that can share their knowledge with their students through the improvement of their training.

This category consists of three subcategories: current training program; communication skills; and barriers to training.

#### 3.2.1 Current training program

The current training program for tutors, although it is not mandatory, was sometimes perceived as too homogeneous, although necessary to acquire a minimum common training basis. The homogenization of the contents was seen as an important aspect.

“*Sometimes you go on a course that you might not have chosen, but it's OK. I think that we all have to attend all the courses and that in the end we all have to have the same training so that there is a balance between the different centers and all the professionals are trained in the same way” (tutor 9)*.

#### 3.2.2 Communication skills

Communication skills were seen as a main axis of the training needs expressed by tutors, not only for teaching, but also to manage conflict situations and being able to work with emotions and attitudes, seeking for the personalization of the training, but avoiding a paternalistic approach.

“*Teaching techniques, communication techniques. Very important to know how to communicate” (tutor 25)*.

A second tutor also added:

“*The most important training need I detect is in relation to how to handle conflict situations” (tutor 23)*.

#### 3.2.3 Barriers to training

Tutors gave a special relevance to the time required for training, considering the great pressure of providing healthcare and the underlying professional exhaustion which affects their own motivation as tutors for the lack of focus on the educational side of their role.

“*Knowing how to combine the healthcare line of action with the space to be able to receive training” (tutor 17)*.

“*You have to stop attending patients to do training and the next day you find yourself with a backlog of work” (tutor 22)*.

### 3.3 Characteristics of an ideal training program

Our informants expressed the main features of an ideal training program that would suit their training needs and help overcome the barriers they encounter. Three main subcategories were identified: main training program content; optimal regulation and planification of the training; and creating a tutors' network.

#### 3.3.1 Main training program content

First of all, elements stand out in relation to the tutoring process, mainly related to communication which reaffirm the training needs previously expressed by the clinician tutors. The current training lacks aspects that arise in the relationship with the students, which are crucial for securing an optimal learning process.

Tutors expressed that the future program should include elements in order to enhance their capability of managing the emotional side of the relationship with the student.

“*I think it is essential to know how to manage the emotional relationship with the student” (tutor 9*).

Tutors also described a need to consider the inclusion of tools or strategies on how to handle “difficult” situations with the students entailing the resolution of potential conflicts.

“*The tutor-resident-patient triangle, the part of how to resolve conflicts is very relevant” (tutor 3)*.

Secondly, tutors highlighted the need to include new topics that are not clinically based such as bioethics, research or new technologies. Bioethics or research, for example, are important aspects in clinical practice, and avoiding them can have a strong impact not only in the clinical practice of tutors, but more specially on how future specialists might consider and apply these aspects in their future clinical practice.

“*I include bioethics, which is important and we often overlook” (tutor 11)*.

“*I have already said that personally, in research I would like to have a little more training” (tutor 25)*.

Moreover, tutors shared their views on the importance of being trained in how to optimally teach and evaluate the students considering the pedagogical perspective of teaching and assessment. The fact that tutors combine clinical and teaching practice entails the need to switch the focus from patient to student, but still following an evidence-based approach given by proven pedagogical tools and strategies.

“*It would be good to have theory about teaching, because we are teachers, we have a colleague who is training at our side and each one trains their student based on their experience and being flexible according to the student's profile” (tutor 26)*.

The heterogeneity of the criteria when facing the evaluation moments was also highlighted. Tutors express they are aware that evaluation is a critical and a difficult moment for the students, but also for them. Enhancing their knowledge on how to optimally evaluate the students and a clear understanding of the evaluation process is key.

“*Evaluation is another handicap and we can address the issue of evaluation to generate knowledge among tutors. Unify criteria” (tutor 17)*.

#### 3.3.2 Optimal regulation and planification of the training

The need for regulating and optimally planning the future programs of tutor training courses was manifested. The future program should consider the responsibility and impact of the training of future specialists, locating teaching at the same level as clinical practice which entails giving space for teaching and training in the tutors' agendas.

“*It would be a good idea to consider a yearly training calendar, structured and well-regulated [to be held] on specific days when at your center attending the training is totally guaranteed” (tutor 5)*.

A second tutor added:

“*It is important that training is developed in a regulated way, the issue of teaching must be seen as a priority in the healthcare centers so that the centers respect those times and we can have training, more than volume in quality”(tutor 5)*.

#### 3.3.3 Creating a tutors' network

Finally, the importance of seeking spaces for clinician tutors to meet is highlighted, that also considers the reception plan of new tutors. Sharing difficulties and experiences was seen as an important element that should be incorporated in the new training plan for tutors. In fact, at the end of the focus groups many tutors expressed their gratitude to the research team for having created a space to share their views and needs, and be willing to design, based on those, a future training plan.

“*What I think is most useful are the meetings between teaching coordinators or when we meet colleagues from other primary healthcare centers” (tutor 13)*.

## 4 Discussion

Our research provides a vision of the experiences and perceptions of clinician tutors related to their own teaching performance and training needs. The main categories identified were: (1) teaching performance; (2) training needs; and (3) characteristics of an ideal training program. The sample consisted of a majority of tutors of medicine compared to nursing, being proportional to the population of tutors accredited at the training unit. Most of the tutors were new to their teaching role.

In relation to training needs, participants require training in communication skills as well as the resolution of challenging situations. A well defined relationship between tutors and students is essential in order to integrate new knowledge, considering a meaningful learning approach and the profile of the students, who are no longer undergraduate students, but already professionals who want to develop a more in depth career path through the clinical specialty postgraduate education (26, 27). In this sense, communication and conflict resolution techniques are essential and constitute precious pedagogical tools, most especially in a mentorship relation in which communication and confidence are key (28, 29). This idea also takes into account an additional reflection of the clinician tutors in regards to the personalization of the training. Mentoring practices for master students in healthcare have demonstrated that mentorship is always necessary, and not only part of the undergraduate teaching-learning process. New professionals also need role models and feedback to advance (30–32).

In terms of the barriers identified by the clinician tutors for the performance of their role and their training, one of the most highlighted ideas was the balance between performance/training and the workload they are exposed to in the clinical sites. Although the training of new specialists is a pressing issue, healthcare systems are extremely tense and clinician tutors struggle to find time and motivation to be trained (33, 34). Healthcare institutions should facilitate and protect mentorship practices by providing time for the training of new tutors as well as the development of contracts that include revenues to clinician tutors for their work as mentors (35, 36). In this regard, teaching units should take these needs into account and advocate respecting the time for training, combining it with clinical, community, teaching, and research activities integrated into the clinician tutor's working day (36).

The content of the training programs was another aspect that was highlighted. Clinician tutors are aware of the need to homogenize the content of the training pathways of the students, and demand learning and discussion forums to share their experiences and queries (37). Regarding the teaching methodologies and its prioritization, those related to feedback, the review of medical records and guidelines, structured observation, video recording, clinical sessions, case studies, self-auditing and reflective thinking stand out. The learning process of the postgraduate training is mainly practical, consequently related strategies to experiential learning such as reflective thinking and continuous feedback should stand out (28, 31, 32). However, historically, in clinical learning structured observation or review of medical records are still very predominant, mainly because of the lack of pedagogical strategies from tutors and protected time to develop those strategies (38). Other approaches, such as problem-based learning, simulators, records auditing, patient opinions, or the opinion of team mates are less used and should be also taken into account in the clinical learning environment (4, 8–10, 19, 39, 40). The use of one or another methodology is also conditioned by the particular characteristics of the residents and clinician tutors, their prior experience, as well as the time available and the physical space to carry them out. This is in line with the literature consulted, which points out that mentoring requires effort, interest, dedication and time and that the lack of resources and/or specific hours for training can affect teacher-student motivation and the quality of tutoring and training (19). This leads one to consider the need to offer a training program that links the skills for developing with professional activities and with specific tasks associated with work contexts, using appropriate methodologies for the acquisition of skills (37, 38).

Finally, in respect of the content of an ideal training plan, aspects are included that can be split in relation to the performance of the teaching role in the tutoring and evaluation spaces such as communication skills, managing emotions, and conflict resolution. In this sense, the evaluation space was also highlighted as a key aspect which can lead to a good final integration of competencies. The process of tutoring is essential and should lead to no surprises in the final evaluation if a constant feedback process has been developed (22, 39, 40). Most of the conflicts between tutor and student may arise in the evaluation spaces which should be scheduled and normalized as part of the learning process (40). The integration of these aspects, both for clinician tutors and students, are essential and should be part of the teaching plan. In terms of the organization of the teaching, the clinician tutors consider that the creation of a tutor network may be a good space for them to meet and discuss clinical and tutoring aspects. The existing scientific literature agrees with this content plan and adds quality management, in addition to guiding the format of training (e.g., online or blended learning) that best suits their needs, as well as establishing synergies, inter-center activities and agreements between the Autonomous Communities and other suppliers (18, 39, 41).

### 4.1 Limitations

This study is not without its limitations. Its main limitation is that we included only one teaching unit of the specialties of family and community nursing and medicine. Additionally one third of the tutors were new and had little experience. By their very nature, the results cannot be extrapolated to other teaching units.

## 5 Conclusions

Specialized health training clinician tutors of family and community nursing and medicine have a great responsibility in the performance of their teaching role and their training is key to the teaching-learning process of future specialists. Clinician tutors express the need to acquire pedagogical tools, to develop communication skills and to create a tutor network in order to improve their mentorship practice. Furthermore, more institutional recognition and protected time are also highlighted as important elements for their mentorship role. The findings of our research can serve as a guideline to start designing a training plan that meets the real needs of clinician tutors.

## Data Availability

The raw data supporting the conclusions of this article will be made available by the authors, without undue reservation.
